# Elongin A regulates transcription *in vivo* through enhanced RNA polymerase processivity

**DOI:** 10.1074/jbc.RA120.015876

**Published:** 2020-12-13

**Authors:** Yating Wang, Liming Hou, M. Behfar Ardehali, Robert E. Kingston, Brian D. Dynlacht

**Affiliations:** 1 Department of Pathology, New York University School of Medicine, New York, New York, USA; 2 Department of Molecular Biology, Massachusetts General Hospital, Boston, Massachusetts, USA; 3 Department of Genetics, Harvard Medical School, Boston, Massachusetts, USA

**Keywords:** Elongin A, RNA polymerase II, transcription, transcription elongation, enhancer, 4sU, 4-thiouridine, 4sU-seq, 4sU-labeled RNA sequencing, ChIP, chromatin immunoprecipitation, CUT&RUN, cleavage under targets and release using nuclease, DRB, 5,6-dichloro-1-beta-D-ribofuranosylbenzimidazole, eRNA, enhancer RNA, ES, embryonic stem, FPKM, fragments per kilobase per million mapped reads, RNAPII, RNA polymerase II, Ser2, serine 2, TESs, transcript end sites, TSS, transcription start site

## Abstract

Elongin is an RNA polymerase II (RNAPII)-associated factor that has been shown to stimulate transcriptional elongation *in vitro*. The Elongin complex is thought to be required for transcriptional induction in response to cellular stimuli and to ubiquitinate RNAPII in response to DNA damage. Yet, the impact of the Elongin complex on transcription *in vivo* has not been well studied. Here, we performed comprehensive studies of the role of Elongin A, the largest subunit of the Elongin complex, on RNAPII transcription genome-wide. Our results suggest that Elongin A localizes to actively transcribed regions and potential enhancers, and the level of recruitment correlated with transcription levels. We also identified a large group of factors involved in transcription as Elongin A–associated factors. In addition, we found that loss of Elongin A leads to dramatically reduced levels of serine2-phosphorylated, but not total, RNAPII, and cells depleted of Elongin A show stronger promoter RNAPII pausing, suggesting that Elongin A may be involved in the release of paused RNAPII. Our RNA-seq studies suggest that loss of Elongin A did not alter global transcription, and unlike prior *in vitro* studies, we did not observe a dramatic impact on RNAPII elongation rates in our cell-based nascent RNA-seq experiments upon Elongin A depletion. Taken together, our studies provide the first comprehensive analysis of the role of Elongin A in regulating transcription *in vivo*. Our studies also revealed that unlike prior *in vitro* findings, depletion of Elongin A has little impact on global transcription profiles and transcription elongation *in vivo*.

The synthesis of mRNA by RNA polymerase II (RNAPII) requires the highly orchestrated action of a large cohort of factors. During the transcription cycle, general transcription factors recruit RNAPII to promoters to initiate transcription, and the RNAPII complex pauses ∼50 to 100 nt downstream of transcription start sites (TSSs). Pausing is enforced by 5,6-dichloro-1-beta-D-ribofuranosylbenzimidazole (DRB) sensitivity-inducing factor, which includes SPT4 and SPT5, as well as negative elongation factor. The release of paused RNAPII into productive elongation is mediated by positive transcription elongation factor b and cyclin-dependent kinase 12, which phosphorylates DRB sensitivity-inducing factor, negative elongation factor, and serine 2 (Ser2) on the C-terminal domain of the largest subunit of RNAPII (1, 2). Transcription initiation has been recognized as a critical regulatory point during the transcription cycle, but recently, a growing body of evidence suggests that transcription elongation can also be an important rate-limiting step. Although transcription elongation rates on individual genes vary widely (3), maintaining a proper elongation rate is critically important. Recent studies suggest that misregulation of transcription elongation can cause defects in transcription directionality, cotranscriptional chromatin modifications, and mRNA splicing (4, 5). A mouse strain harboring a slow RNAPII mutant shows embryonic lethality, suggesting that proper RNAPII elongation is also critical for mouse development (6).

Several factors have been shown to mediate proper transcription elongation, including PAF1 complex, SPT6, and SPT5 (7, 8, 9). Another factor that is potentially involved in regulating RNAPII elongation is the Elongin complex (10), which was identified biochemically as one of the factors that can stimulate RNAPII elongation on DNA templates *in vitro*. Previous studies suggested that Elongin can stimulate RNAPII elongation *in vitro*, potentially by overcoming the transient pausing of RNAPII on a DNA template. However, the role of this complex during transcription elongation has not been tested *in vivo*.

The Elongin complex is a trimer consisting of Elongin A, B and C, and Elongin A is the largest subunit. The N-terminus of Elongin A shares homology with elongation factor transcription elongation factor IIS, and the C-terminus of Elongin A contains a conserved motif, BC-box, that allows the Elongin B and C subunits to bind. Elongin A is the transcription-stimulatory subunit, and Elongin B and C can enhance the activity of Elongin A (10, 11). Apart from Elongin A, a large group of proteins also contains a BC-box motif and can associate with Elongin B and C. These proteins can be linked to a Cullin-RING ligase through the Elongin BC complex to function as an E3 ligase complex. The Cullin-RING ligase in these cases contains Cul2 or Cul5 and Rbx1 or Rbx2 (12, 13, 14). Upon UV irradiation, the mammalian Elongin complex can assemble with the Cul5/Rbx2 module to act as the ubiquitin ligase for RNAPII subunit, RPB1 (15). It was also shown that the yeast homologs of Elongin A and C are responsible for the ubiquitination of RNAPII under conditions of DNA damage (16).

The Elongin complex was initially identified as a potential elongation factor in *in vitro* studies more than 2 decades ago. Since then, several studies have been performed to investigate the role of Elongin *in vivo*. It was shown that Elongin A is required for transcriptional induction in response to multiple stimuli in mammalian cells (17, 18), and Elongin A–deficient mouse embryo exhibit developmental abnormalities (18). Cellular immunofluorescence studies suggest that Elongin A colocalizes with the hyperphosphorylated form of RNAPII, and chromatin immunoprecipitation (ChIP)-quantitative PCR experiments show Elongin A associates with stress-response genes upon induction (17). However, although it has been generally assumed that Elongin also functions as a transcription elongation factor *in vivo*, none of these prior studies has confirm this hypothesis. In addition, there have been no genome-wide studies of Elongin, and the impact of Elongin on different aspects of the transcription cycle has not been systematically investigated. Here, we investigated the genome-wide localization of Elongin A and the impact of its loss on RNAPII distribution, as well as the production of nascent and mature transcripts. Our studies suggest that Elongin A localizes to actively transcribed genomic regions, including annotated transcripts and potential enhancer regions. Concordantly, we found that Elongin A associates with a large group of factors involved in transcription. Interestingly, we showed that Elongin A depletion has a dramatic impact on levels of the Ser2-phosphorylated form of RNAPII and a less dramatic impact on Ser5 and total RNAPII levels. Importantly, our study also revealed that unlike prior *in vitro* findings, Elongin A depletion has little impact on transcription elongation rates *in vivo* genome-wide.

## Results

### Elongin A localizes to actively transcribed regions

We first attempted to identify Elongin A binding sites genome-wide by ChIP-seq. However, the enrichment was not efficient, and after peak-calling, we identified 186 peaks (Fig. S1*A*). To overcome this obstacle, we used an alternative approach, cleavage under targets and release using nuclease (CUT&RUN) (19, 20). To improve Elongin A enrichment, we modified the original protocol by fixing the cells with formaldehyde to stabilize the chromatin structure. We performed CUT&RUN in DLD1, a human colon cancer cell line, with anti–Elongin A antibody (Fig. S1*B*) and immunoglobulin G (IgG) controls. CUT&RUN allowed for a robust signal-to-noise ratio, resulting in the reproducible identification of 8720 Elongin A binding peaks (Fig. S1*A*). After comparison with Refseq annotations, we found that these peaks corresponded to Elongin A occupancy of 2064 genes. Molecular pathway analysis of Elongin A occupied genes showed that ribosomal proteins, mRNA processing, ciliary, and VEGF signaling pathway proteins, as well as translation factors are among the top enriched categories (Fig. S1*C*).

**Figure 1:**
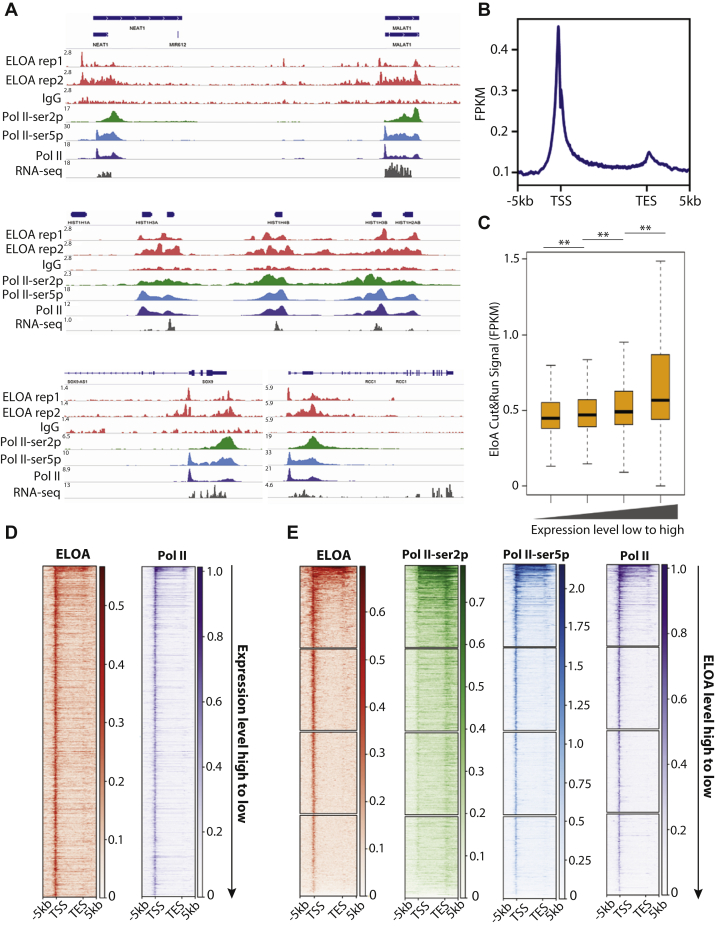
**Characterization of the Elongin A biding site genome-wide by CUT&RUN.***A*, representative screenshots showing Elongin A CUT&RUN enrichment at transcribed regions and its colocalization with RNAPII and RNA-seq signal. *B*, the metaplot of average Elongin A CUT&RUN signal on 4544 expressed, nonoverlapped Refseq genes from TSS to TES. *C*, correlation between Elongin A signal and gene expression. *D*, heat maps of Elongin A and RNAPII signal on 4544 expressed, nonoverlapped Refseq genes. Genes were ranked by their expression level (high to low). *E*, heat maps of Elongin A and RNAPII–ser2p, pol II–ser5p, and total pol II signal on 4544 expressed, nonoverlapping Refseq genes. Genes were ranked by Elongin A occupancy (high to low). CUT&RUN, cleavage under targets and release using nuclease; RNAPII, RNA polymerase II. ∗∗ stands for *p* < 0.05.

Next, we performed RNA-seq and RNAPII ChIP-seq analysis and compared these results with our Elongin A occupancy data. We found that Elongin A was enriched on actively transcribed genes and binding was highly correlated with active transcription (Fig. 1*A* and Fig. S1*D*). We found that ChIP-seq peaks were also observed over some regions that exhibited strong Elongin A enrichment in CUT&RUN experiments (Fig. S1*D*). Metagene analysis of annotated Refseq genes suggests that Elongin A is enriched at TSS regions, gene bodies, and transcript end sites (TESs) (Fig. 1*B*). Interestingly, we found that we were more likely to observe Elongin A enrichment on gene bodies and regions downstream of the TES on short genes, whereas on longer genes, we were more likely to observe Elongin A enrichment only at TSS-proximal regions (Fig. 1*A*, Fig. S1*E*). These results suggest that Elongin A may only travel with the RNAPII complex up to a certain distance downstream of TSS or that Elongin A has more important roles near TSS. Interestingly, when we plotted Elongin A CUT&RUN signal in the immediate vicinity of the TSS, we found that Elongin A enrichment is stronger immediately upstream of the TSS than downstream (Fig. S1*F*). Our analysis also suggested that Elongin A enrichment correlated with gene expression levels. We ranked Refseq genes based on their expression levels (fragments per kilobase per million mapped reads [FPKM]) and divided them into four groups. We then quantified average Elongin A enrichment on each group of genes and found that genes with higher expression levels tended to show stronger Elongin A enrichment (Fig. 1*C*). Plotting individual genes on heat maps likewise showed a clear correlation between gene expression and Elongin A enrichment (Fig. 1*D*). We also ranked Refseq genes on heat maps based on their Elongin A enrichment and simultaneously plotted enrichment of RNAPII on individual genes. The results suggest that genes with greater Elongin A enrichment also tend to be more highly transcribed (Fig. 1*E*). Our results therefore provide the first genome-wide maps of Elongin A occupancy and indicate a clear correlation between the binding of this factor and gene expression levels. These observations are consistent with results from an independent study (21).

### Elongin A localizes to potential enhancers

**Figure 2:**
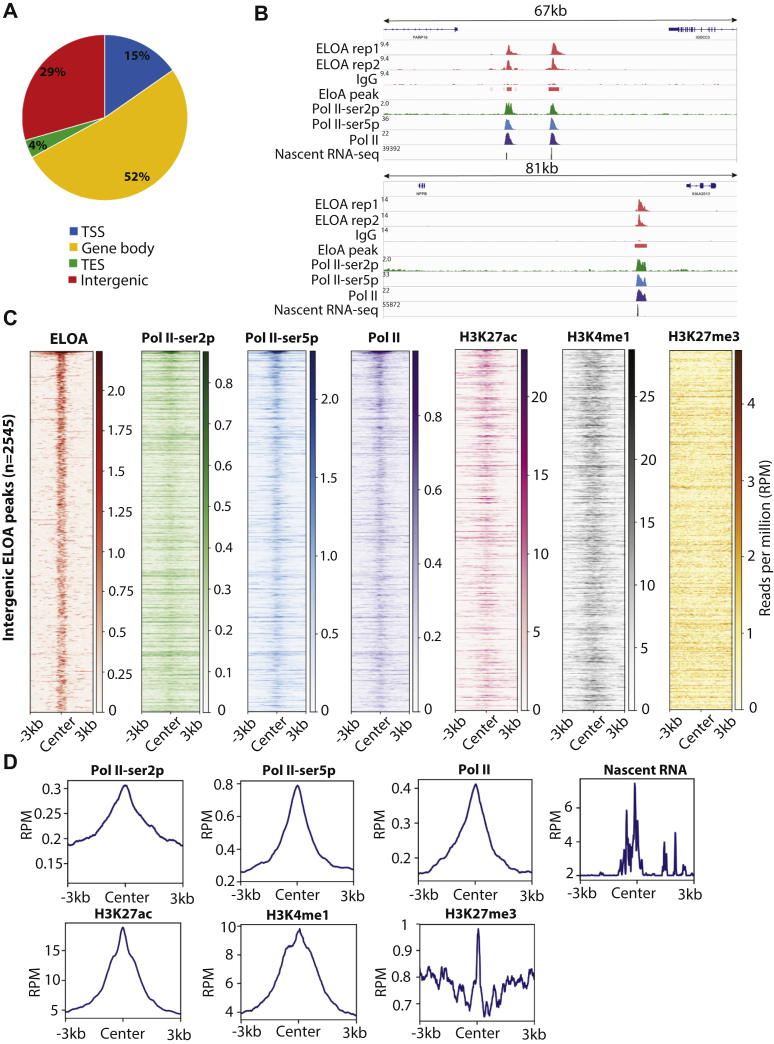
**Elongin A occupies intergenic regions that are potential enhancers.***A*, annotation of Elongin A peaks. *B*, representative screenshots showing Elongin A CUT&RUN signal at intergenic regions and its colocalization with RNAPII and nascent transcripts. *C*, heat maps of different ChIP-seq signals on 2545 intergenic Elongin A peaks. Heat maps were centered on the summit of the Elongin A peak. *D*, the metaplot of different ChIP-seq and nascent RNA-seq signals on the 2545 intergenic Elongin A peaks. Plots were centered on the summit of the Elongin A peak. CUT&RUN, cleavage under targets and release using nuclease; RNAPII, RNA polymerase II; TESs, transcript end sites.

Analysis of our Elongin A binding data suggests that ∼30% of the peaks localize to intergenic regions (Fig. 2*A*, Fig. S2*B*), and this observation prompted us to further investigate these binding events. First, we investigated whether these regions were actively transcribed by analyzing nascent transcription genome-wide using a pulse of 4-thiouridine (4sU) and high-throughput sequencing (4sU-seq). Interestingly, many of the intergenic peaks bound by Elongin A also exhibited prevalent nascent transcription (Fig. 2*B*), and at some regions, the transcription activity appears to be bidirectional (Fig. S2*A*). Thus, we speculated that these regions could be active enhancers. We also compared these intergenic peaks with RNAPII ChIP-seq data and observed strong enrichment for Pol II over these peaks (Fig. 2, *B*–*D*). Because ChIP-seq data are not publicly available for DLD1 cells, we took advantage of ChIP-seq data for H3K27ac and H3K4me1 in HCT116, another widely used colon cancer cell line, available through ENCODE, because enhancers tend to be conserved in a lineage-specific and cell type–specific manner. We compared these two enhancer marks with intergenic Elongin A peaks and observed strong enrichment for H3K27ac and H3K4me1 near these intergenic peaks (Fig. 2, *C*–*D*, and Fig. S2*C*). In contrast, a repressive mark, H3K27me3, did not show any enrichment at these peaks (Fig. 2, *C*–*D*, and Fig. S2*C*). Although DLD1 cells do not have enhancer annotations available, our results suggest that the intergenic Elongin A is recruited to potential enhancer regions and that Elongin A may participate in the transcription of enhancer RNAs (eRNAs). Importantly, independent studies in mouse embryonic stem (ES) cells, which have well annotated enhancers, confirmed that Elongin A localizes to enhancers (21).

### Elongin A associates with factors related to transcription

**Figure 3:**
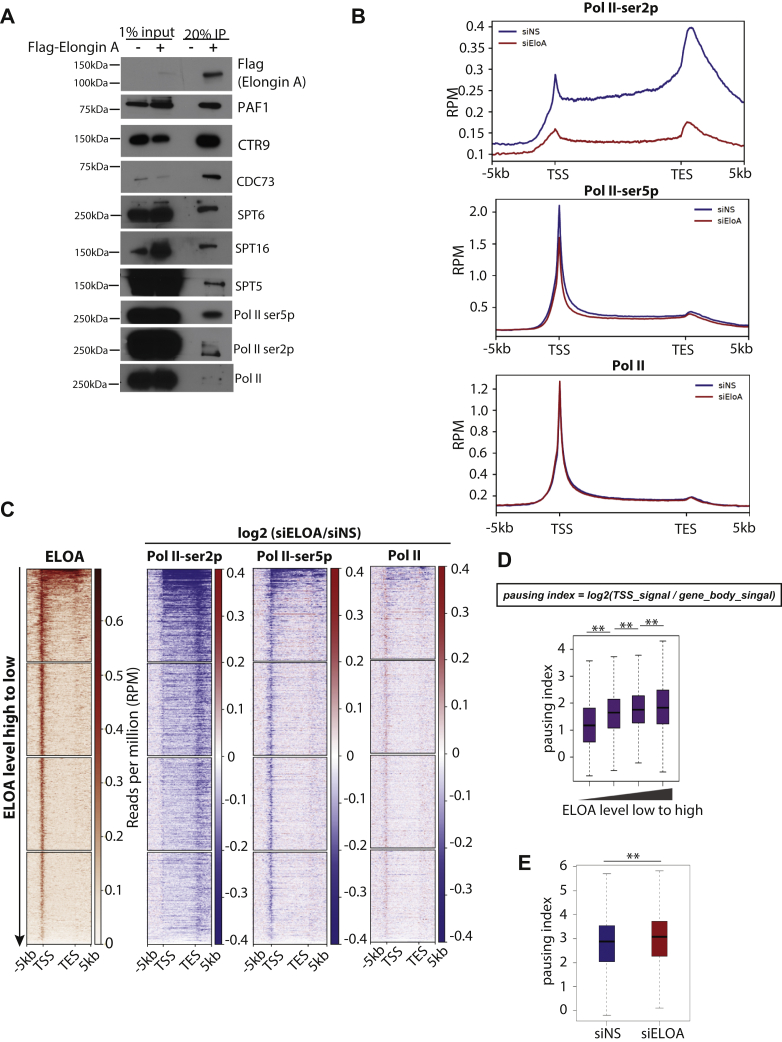
**Elongin A associates with a group of transcription elongation/processing factors and regulates RNAPII.***A*, Western blots validating Elongin A–interacting partners identified by mass spectrometry in DLD1 cell. *B*, the metaplot of average ChIP-seq enrichment on 4544 expressed, nonoverlapped Refseq genes in control (siNS) and Elongin A–depleted (siEloA) cells. *C*, heat maps of the log_2_ fold change upon Elongin A depletion for each ChIP-seq on 4544 expressed, nonoverlapped Refseq genes. Genes were ranked by Elongin A occupancy (high to low). Note that negative values (*blue*) indicate a reduced signal upon Elongin A depletion. *D*, correlation between the Elongin A signal and pausing index. *E*, the pausing index in control and Elongin A–depleted cells. ChIP, chromatin immunoprecipitation; RNAPII, RNA polymerase II.

In an effort to explore regulatory roles for Elongin A, we sought to screen for its associated proteins. We constructed a DLD1 cell line expressing Flag-tagged Elongin A at endogenous levels and performed a screen for interacting proteins using anti-Flag immunopurification of solubilized chromatin. We analyzed Elongin A–interacting partners by mass spectrometry and identified a large group of Elongin A–associated proteins (Fig. S2, *D*–*E*). We confirmed a subset of interacting proteins by immunoprecipitation and Western blotting (Fig. 3*A*, Fig. S2*E*). Consistent with previous studies, we identified RNAPII subunits and found that a large number of peptides originated from proteins related to transcription elongation and RNA processing. Among the top hits, we identified nearly all subunits of the PAF1 complex, except for RTF1, which does not stably associate with mammalian PAF1 (22). Concordantly, in our previous PAF1 proteomic study, Elongin A was also among the top hits (7), and studies from Ardehali *et al.* (21) also revealed stable interactions between Elongin A and PAF1 complex subunits, attesting to their conserved interactions across species and cell lines. We also identified several subunits of the Integrator complex, a multisubunit complex that has been shown to participate in eRNA processing (23), a finding consistent with our conclusion that Elongin A localizes to potential enhancers. Collectively, our results suggest that Elongin A stably interacts with RNAPII and the transcription machinery on chromatin.

### Loss of Elongin A affects phosphorylated RNAPII

Given that Elongin A associates with RNAPII and a large group of elongation/processing factors, we asked how Elongin A modulates RNAPII transcription after successfully depleting Elongin A with an siRNA pool (Fig. S3*A*). We first performed RNAPII ChIP-seq in control and Elongin A–depleted cells, and we monitored total RNAPII as well as Ser5- and Ser2-phosphorylated forms. Ser5 first appears during the initiation phase at the TSS and is most strongly enriched over early gene bodies, whereas Ser2 phosphorylation correlates with elongating polymerase and is more highly enriched on gene bodies (1). Although we did not observe significant changes in the total RNAPII distribution upon Elongin A depletion, Ser2-phosphorylated RNAPII was dramatically reduced upon Elongin A depletion. Ser5-phosphoylated RNAPII level also diminished after Elongin A depletion, although to a lesser extent than the Ser2-phosphorylated form (Fig. 3*B*, Fig. S3, *B*–*C*). When we ranked Refseq genes based on their Elongin A enrichment, we found that genes with higher Elongin A occupancy also exhibited stronger reductions in phosphorylated RNAPII (Fig. 3*C*, Fig. S2*D*). We also observed a similar pattern at intergenic Elongin A peaks (Fig. S2*E*).

Given that (1) we observed strong reductions in ChIP-seq experiments detecting enrichment of Ser2-phosphorylated, but not total, RNAPII (which primarily corresponds to the unphosphorylated form binding proximal to TSS regions) (Fig. 3*B*) in Elongin A–depleted cells, and (2) Elongin A is highly enriched near the TSS, we examined the relationship between Elongin A enrichment and RNAPII pausing. To address this, we used RNAPII ChIP-seq data to calculate the “pausing index,” defined as the ratio of promoter signal to gene body signal. We then ranked, and divided into four groups, all Elongin A–bound genes based on the levels of factor recruitment and compared Elongin A enrichment with the pausing index. We found that genes with stronger Elongin A enrichment tended to have a higher pausing index (Fig. 3*D*), consistent with independent observations in mouse ES cells (21). Next, we examined the impact of Elongin A depletion on pausing. We found that there was increased pausing in Elongin A–depleted cells (Fig. 3*E*). These results suggest that Elongin A may participate in the release of paused RNAPII into a productive elongation complex.

### Loss of Elongin A did not significantly alter gene expression

**Figure 4:**
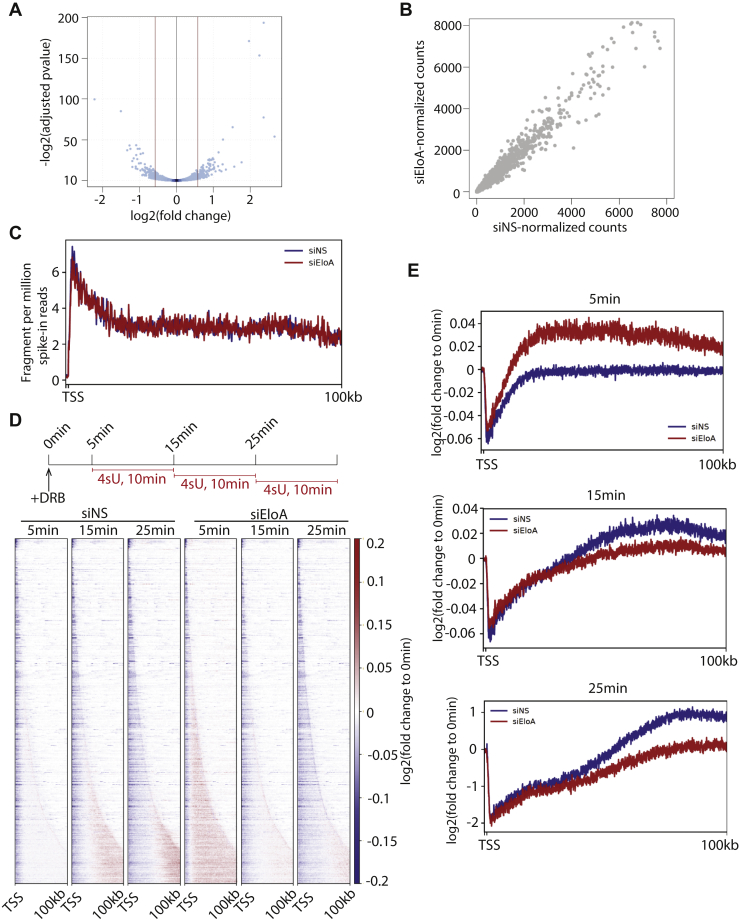
**Impact of Elongin A depletion on gene expression and elongation rates.***A*, volcano plot representing RNA-seq results in control and Elongin A–depleted cells indicating that a small number of genes were differentially expressed. *B*, the dot plot showing the DESeq2-normalized read counts of 4sU-seq in control (siNS) and Elongin A–depleted cells (siEloA). We failed to identify differentially expressed genes in 4sU-seq. *C*, the metaplot of 4sU-seq signal in control (siNS) and Elongin A–depleted cells (siEloA). *D*, *Top*, the scheme of the experimental design to measure the elongation rate. *Bottom*, heat maps of log_2_ fold change at each a time point compared with 0 min. Note that negative values (*blue*) indicate clearance of reads. 4544 expressed, nonoverlapping Refseq genes were ranked by the length (short to long). *E*, metaplot of log_2_ fold change at each time point compared with 0 min. Note that at 5 min, the *red line* (siEloA) is closer to TSS, suggesting that there may be a delay in the clearance of 4sU signal. 4sU, 4-thiouridine.

We then asked whether loss of Elongin A alters the transcriptome and performed RNA-seq on control and Elongin A–depleted cells. Our analyses revealed that a relatively small group of genes exhibited a significant change (fold change >1.5, q-value < 0.1) upon Elongin depletion (Fig. 4*A*, Fig. S4*A*), as 264 genes were upregulated, and 257 genes were downregulated. To monitor nascent transcription, we also performed RNA-seq using a short 4sU pulse (10 min). Analysis with 4sU-seq suggested that, overall, there were no significant or consistent changes in nascent transcription profiles in Elongin A–depleted cells as compared with controls (Fig. 4*B*, Fig. S4*B*). In addition, we did not observe significant changes in nascent transcription levels near the TSS (Fig. 4*C*, Fig. S4*C*), suggesting that transcription initiation is not affected in the absence of Elongin A.

Prior studies suggested that Elongin A stimulates the RNAPII elongation *in vitro* (10). However, whether Elongin A is also critical for RNAPII elongation *in vivo* has not been investigated. Our proteomic screen identified a group of elongation factors as Elongin A–associated proteins. This prompted us to ask whether Elongin A also affected RNAPII elongation rates *in vivo*. To this end, we treated cells with DRB and performed 4sU-seq at different time points (Fig. 4*D*). Because DRB inhibits the release of paused RNAPII into a productive elongation complex, this method allowed us to track the clearance of existing elongating RNAPII complexes over gene bodies with time. We plotted the signal at each time point as a function of the 0-min time point (no DRB). Thus, a negative log_2_ value suggests decreased signal (clearance). Our analysis suggested that this method is robust, as we can clearly observe clearance of 4sU signal from the TSS with time (Fig. 4, *D*–*E*). Although we observed modest delays in 4sU clearance at some early time points (5 min) upon Elongin A depletion (Fig. 4*E*, Fig. S4*E*), the effect did not appear to be dramatic. More importantly, we did not observe similar delays upon Elongin A depletion at other time points. (Fig. 4, *D*–*E*, Fig. S4, *D*–*E*). These results suggest that Elongin A does not significantly enhance RNAPII elongation rates, as it does under certain *in vitro* conditions (10). Importantly, studies in mouse ES cells yielded similar results (21), suggesting that Elongin A is not a major factor for regulation of elongation rates *in vivo*.

## Discussion

The Elongin complex was one of the first complexes identified to be required for proper RNAPII elongation rates *in vitro* more than 2 decades ago, yet its impact on RNAPII elongation and transcription *in vivo* has not been explored. Here, we provide a comprehensive analysis of the impact of Elongin A on RNAPII elongation and transcription genome-wide. We note that although our studies were in progress, independent work using mouse ES cells provided results that largely recapitulated the findings in our study (21).

Prior studies by Kawauchi *et al.* (17) showed using immunofluorescence that Elongin A colocalizes with newly synthesized RNA, and ChIP-quantitative PCR experiments suggested that Elongin A occupied genes induced upon stress treatment. Here, we used a newly established method, CUT&RUN, to map Elongin A binding sites genome-wide in unperturbed mammalian cells. Our results suggest that Elongin A localizes to actively transcribed regions and potential enhancer regions, and we found that Elongin A enrichment positively correlated with gene expression levels. We observed the strongest Elongin A enrichment near TSS regions and also observed enrichment within gene bodies and regions downstream of TESs. Elongin A enrichment appears to be higher at TSS-proximal regions than other genic regions. Interestingly, a closer inspection of the distribution of Elongin A near the TSS suggests that Elongin A could be loaded preferentially upstream of the TSS rather than downstream (Fig. S1*F*). This enrichment is different from the canonical RNAPII profile, wherein RNAPII is skewed toward regions downstream of the TSS. This could suggest that Elongin A plays a more important role in antisense transcription or that Elongin A is loaded onto genes before RNAPII. However, we were not able to detect changes in antisense transcription or transcription directionality with our 4sU-seq data (data not shown). Our immunoprecipitation results suggest that Elongin A associates more robustly with RNAPII–Ser5p than with RNAPII–Ser2p (Fig. 3*A*). However, RNAPII ChIP-seq results suggest that Elongin A–depleted cells show enhanced promoter RNAPII pausing and dramatically reduced RNAPII–Ser2p levels (Fig. 3, *B* and *E*). In addition, we found that genes with stronger Elongin A enrichment also tended to have a higher pausing index (Fig. 3*D*). These results suggest that Elongin A is potentially involved in the release of paused RNAPII into productive elongation complexes. This is consistent with the fact that Elongin A is more highly enriched at the TSS than other genic regions. Accordingly, we observe strong reductions in the level of RNAPII–Ser2p genome-wide upon Elongin A depletion, whereas the levels of total RNAPII did not change significantly.

Our CUT&RUN results showed that many intergenic regions exhibit strong Elongin A enrichment. Closer examination of these intergenic regions revealed that these regions are also enriched for RNAPII and nascent transcripts, suggesting that these regions are potential enhancers. Furthermore, by examining nascent transcription, we found that the transcription from some of these regions is bidirectional (Fig. S2*A*), which is a feature of eRNA (24). Although enhancers have not been annotated in DLD1 cells, data from HCT116, another colon cancer cell line, showed that these Elongin A–occupied intergenic regions are enriched for enhancer histone marks. These results indicate a possible role for Elongin A at enhancers. However, whether Elongin A participates in the transcription of eRNA and/or 3D chromatin architecture remains to be investigated.

*In vitro* studies suggest that Elongin A can stimulate RNAPII elongation on a DNA template. However, we did not observe a dramatic impact on RNAPII elongation rates in Elongin A–depleted cells *in vivo*. It should be noted that prior *in vitro* studies were performed under conditions where limiting factors and low concentrations of NTP were provided. Under such conditions, Elongin A can dramatically reduce the time for full-length transcripts to appear. It would be difficult to recapitulate such conditions *in vivo*, and the *in vitro* method did not allow one to track the movement of RNAPII along the entire gene over time. Our data for the first time suggest that Elongin A does not stimulate RNAPII elongation rates *in vivo*. Alternatively, Elongin A may be a positive regulator of RNAPII elongation, but other elongation factors can compensate to modulate elongation rates in its absence. Notably, apart from Elongins A, B, and C, the human Elongin family includes two other members, Elongin A2 and Elongin A3. Both Elongins A2 and A3 share structural similarity with Elongin A, can form complex with Elongin BC, and can stimulate transcription *in vitro* (25, 26). It remains possible that one or both of these proteins can compensate for the absence of Elongin A.

In our screen for Elongin A–interacting partners, we found that subunits of the PAF1 complex were among the top hits, and in our prior PAF1 proteomic study, Elongin A was also among the top hits, suggesting strong and stable interactions between the two complexes. Our Elongin A and Paf1 proteomes share many common hits, such as the FACT complex, SPT5, SPT6, Integrator complex, and others. However, Elongin A and PAF1 seem to affect transcription in very different ways. For example, we observed dramatic decreases in RNAPII elongation rates and accumulation of RNAPII on genes in Paf1-depleted cells (7). In Elongin A–depleted cells, however, we did not observe dramatic changes in the elongation rate, whereas the RNAPII–Ser2p level was dramatically reduced. We find it unlikely that these differences can be explained by cell-type differences, although currently, we cannot formally rule out this explanation. Rather, Elongin A and PAF1 may regulate transcription in a very different manner, and although these proteins are likely to exist in the same complex, they may not function synergistically.

In addition to the PAF1 complex, we also captured several other factors involved in transcription elongation, suggesting that Elongin A may associate with elongating polymerase. However, Elongin A does not appear to affect RNAPII elongation significantly, suggesting it may have a role in fine-tuning transcription. Interestingly, we also captured many splicing factors in our experiment, suggesting that Elongin A may be involved in mRNA processing, although further studies are needed to validate whether Elongin A affects transcript processing.

Previous studies suggested that Elongin A is required for transcriptional induction of certain genes in response to different stimuli. Here, we did not observe many differentially expressed genes upon Elongin A depletion in DLD1 cells under normal culture conditions. Even when we set the fold-change threshold to 1.5-fold, we only observed a small group of differentially expressed genes (Fig. 4*A*), and we also did not observe significant changes in nascent transcription levels or transcription initiation by 4sU-seq. Whether Elongin A is required for transcription induction in response to stimuli *in vivo*, however, needs to be further investigated.

## Experimental procedures

### Cell culture and transfection

DLD1 cells were purchased from ATCC (CCL-221) and grown in RPMI (Corning) supplemented with 10% fetal bovine serum and 1% penicillin and streptomycin. Elongin A siRNA was purchased from Horizon Discovery (siGENOME SMARTPool, M-005143-03). All siRNAs (final concentration 50 nM) were delivered using Lipofectamine RNAiMax (Invitrogen) as per the manufacturer’s protocol. Cells were harvested 48 h after transfection for downstream analysis.

### ChIP and ChIP-seq

ChIP was performed as previously described (27). Briefly, cells were fixed with 1% formaldehyde for 10 min at RT and quenched with 0.125 M glycine at RT for another 10 min. Cells were then washed with PBS, scraped off the culture plate, and collected by centrifugation. Cell pellets were first treated with ChIP buffer 1 (50-mM Hepes, pH 7.5, 140-mM NaCl, 1-mM EDTA, 10% glycerol, 0.5% NP-40, and 0.25% Triton X-100) for 10 min at 4 °C. Lysates were centrifuged at 4 °C, 4000 rpm for 10 min, and the supernatant was discarded. The pellets were then treated with ChIP buffer 2 (10-mM Tris, pH 8.0, 200-mM NaCl, 1-mM EDTA, and 0.5-mM EGTA) at RT for 10 min. Lysates were centrifuged at 4 °C, 4000 rpm, for 10 min, and the supernatant was discarded. The pellets were resuspended in ChIP buffer 3 (10-mM Tris, pH 8.0, 1-mM EDTA, 0.5-mM EGTA, and 0.5% sodium sarkosyl) and sonicated on ice to an average length of 300 bp. Lysates were then centrifuged at 4 °C, 13,000 rpm, for 15 min, and the supernatant (chromatin) was collected and used for ChIP. Twenty five milligram of chromatin was used for each ChIP reaction. Chromatin was precleared with protein A Sepharose beads at 4 °C for 1 h before incubating with 2 μg of antibody overnight. The next day, lysates were incubated with protein A Sepharose beads for 4 h. Beads were washed with a wash buffer (50 mM Hepes pH 7.6, 500 mM LiCl, 10 mM EDTA, 1% NP-40, 0.7% sodium deoxycholate) 8 times, and antibody-bound chromatin was eluted by incubating the beads in an elution buffer (10-mM Tris, pH 8.0, 1-mM EDTA, 1% SDS) at 65 °C for 20 min. All buffers were supplemented with protease and phosphatase inhibitors. ChIP-seq libraries were generated as described (28) and sequenced by paired-end Illumina sequencing.

The following antibodies were used for ChIP in this study: PolII-ser2p (Abcam, ab5095), PolII-ser5p (Abcam, ab5131), and PolII (BioLegend, 664906).

### CUT&RUN

CUT&RUN was performed as described by Skene *et al.* (19) with the following modifications. Cells were fixed with 1% formaldehyde at RT for 10 min and quenched with 0.125 M glycine for another 10 min at RT. Cells were then washed with PBS, and 10^6^ formaldehyde-fixed cells were used for each CUT&RUN reaction. We included 0.1% Tween-20 and 0.1% BSA and removed spermidine from the original wash buffer recipe to prevent clumping of concanavalin A–coated magnetic beads. Digitonin (0.05%) was used for permeabilization and 1.5-μg Elongin A antibody or normal goat IgG was used in each reaction. The final DNA product was extracted with phenol–chloroform and used to make sequencing libraries using methods similar to ChIP-seq library preparation with 14 PCR cycles. The CUT&RUN libraries were sequenced by paired-end Illumina sequencing.

The following antibodies were used in CUT&RUN experiments in this study: Elongin A (Santa Cruz Biotechnology, sc-1557), rabbit anti-goat IgG (Thermo Fisher Scientific, 31105), and goat IgG (Invitrogen, 02-6202).

### Immunopurification and mass spectrometry

WT DLD1 cells or DLD1 cells stably expressing Flag-tagged Elongin A were used for immunopurification of soluble chromatin. To extract the chromatin fraction, cell pellets were first resuspended in solution A (10-mM Hepes, pH 7.9, 10 M KCl, 1.5-mM MgCl_2_, 0.34 M sucrose, 10% glycerol, and 1-mM DTT) supplemented with 0.1% Triton X-100 and incubated on ice for 5 min. The lysate was then centrifuged at 4 °C, 1400*g*, for 5 min. The supernatant was discarded. The pellet (nuclei) was resuspended with buffer A supplemented with 1-mM CaCl_2_ and 5 U/μl MNase and incubated at RT for 30 min. MNase digestion was stopped by adding EGTA to a final concentration 1 mM, and the lysate was centrifuged at 4 °C, 1400*g*, for 5 min. The supernatant was discarded. The pellet was then resuspended in solution B (2-mM EDTA, 0.2-mM EGTA, and 1-mM DTT) and incubated on ice for 30 min. The lysate was then centrifuged at 4 °C, 1700*g*, for 5 min, and the supernatant was collected and used for purification (immunoprecipitation). 1 mg of the chromatin lysate was used for each IP. Hepes, pH 7.6, NaCl, and NP-40 were added to the lysate at a final concentration of 25-mM Hepes, pH 7.6, 150-mM NaCl, and 0.1% NP-40. The lysate was precleared with protein A/G beads for 1 h at 4 °C and then incubated with Flag-M2 beads (Sigma, A2220) for 4 h at 4 °C with rotation. Beads were then washed with a wash buffer (25-mM Hepes, pH 7.6, 150-mM NaCl, and 0.1% NP-40) 5 times. For mass spectrometry, beads were eluted with an elution buffer (0.2-M Tris, pH 7.5, 150-mM NaCl, and 1-mM DTT) supplemented with 2 mg/ml 3X Flag peptide for 45 min at 4 °C. For immunoblots, beads were eluted with Laemmli buffer and boiled for 5 min. All buffers were supplemented with protease and phosphatase inhibitors.

The following antibodies were used for immunoblots in this study: Elongin A (Bethyl Laboratories, A300-943), Flag (Sigma, F1804), PAF1 (Bethyl Laboratories, A300-173), CTR9 (Bethyl Laboratories, A301-395), CDC73 (homemade), SPT6 (Bethyl Laboratories, A300-801), SPT16 (Cell Signaling Technology, 12191), SPT5 (Bethyl Laboratories, A300-868), PolII (BioLegend, 664906), PolII-ser2p (Bethyl Laboratories, A300-654), and PolII-ser5p (Abcam, ab5131).

Mass spectrometry analysis was performed at NYU School of Medicine Proteomics Laboratory. Eluted samples were reduced with DTT and alkylated with iodoacetamide before loaded on NuPAGE 4 to 12% Bis-Tris Gel (Life Technologies). Gel was run for 30 min at 200 V and stained with GelCode Blue Stain Reagent (Thermo Fisher Scientific). The whole lane was excised out and destained with 1:1 mixed methanol and 100-mM ammonium bicarbonate. Destained gel was then dehydrated and digested with trypsin before subjected to peptide extraction. Peptides were gradient eluted from the column directly to the Q Exactive HF-X mass spectrometer (Thermo Fisher Scientific) using a 1-h gradient. High resolution of full mass spectrometry spectra was acquired with a resolution of 45,000, an automatic gain control target of 3e6, a maximum ion time of 45 ms, and scan range of 400 to 1500 m/z. After each full mass spectrometry, twenty data-dependent high-resolution higher-energy collisional dissociation mass spectrometry (HCD MS/MS) spectra were acquired. All MS/MS spectra were collected using the following instrument parameters: resolution of 15,000, automatic gain control target of 1e5, maximum ion time of 120 ms, one microscan, 2 m/z isolation window, fixed first mass of 150 m/z, and normalized collision energy of 27. MS/MS spectra were searched against the UniProt *Homo sapiens* reference proteome database (downloaded 02/2019) containing common contaminant proteins using Proteome Discoverer 1.4.0.288, and 30,993 entries in the database were searched. Sequest HT search engine was used. The search parameters were as follows: precursor mass tolerance ± 10 ppm, fragment mass tolerance ± 0.02 Da, digestion parameters trypsin allowing two missed cleavages, fixed modification of carbamidomethyl on cysteine, variable modification of oxidation on methionine, deamidation on glutamine and asparagine. The threshold for accepting individual spectra was minimum peptide length of 6 and maximum peptide length of 144 with an signal to noise threshold (FT only) 1.5. The data were filtered using a 1% peptide and protein false discovery rate cut-off searched against a decoy database.

### Nascent RNA labeling and sequencing (4sU-seq)

For nascent RNA sequencing experiments in this article, DLD cells were treated with or without 100-μM DRB (Sigma, D1916) for indicated time. Nascent RNA was labeled by adding 1-mM 4sU (Sigma, T4509) for 10 min. Labeling was stopped by adding TRIzol (Thermo Fisher Scientific, 15596018) directly to the cell culture plate, and total RNA was extracted as per the manufacture’s protocol. 100-μg total RNA was used for purification of nascent RNA. *Saccharomyces cerevisiae* RNA labeled with 4sU was used as a spike-in control. 100 μg mammalian RNA was mixed with 1-μg *S. cerevisiae* RNA and fragmented by adding ice-cold NaOH to the final concentration of 0.2 N and incubating on ice for 20 min. Fragmentation was stopped by adding an equal volume of 1 M Tris, pH 6.8, and RNA was cleaned up performing buffer exchange with P-30 Micro Bio-Spin column (Bio-Rad, 7326223). Biotinylation of 4sU-labeled RNA was carried out in biotinylation buffer (10-mM Tris, pH 7.4 and 1-mM EDTA) supplemented with 0.2 mg/ml EZ-Link HPDP-biotin (Thermo Fisher Scientific, A35390) at RT for 2 h with rotation. RNA was then purified by phenol:chloroform extraction, and biotin RNA was enriched by incubating RNA with 50-μl Streptavidin C1 beads (Thermo Fisher Scientific, 65001) in a binding buffer (10-mM Tris, pH 7.4, 300-mM NaCl, and 0.1% Triton X-100) for 20 min at RT with rotation. Beads were then washed with a high-salt buffer (50-mM Tris, pH 7.4, 2 M NaCl, and 0.5% Triton X-100) twice, binding buffer (10-mM Tris, pH 7.4, 300-mM NaCl, and 0.1% Triton X-100) twice, and a low-salt buffer (5-mM Tris, pH 7.4, 0.1% Triton X-100) once. Biotin RNA was eluted twice by directly adding 300-μl TRIzol to beads. RNA was then extracted as per the manufacture’s protocol. Purified RNA was used to make sequencing libraries with a NEBNext small RNA library prep kit for Illumina (NEB, E7300) or NEBNext Ultra II directional RNA library prep kit for Illumina (NEB, E7760). Libraries were sequenced by paired-end Illumina sequencing.

### Bioinformatics

#### ChIP-seq and CUT&RUN analysis

Paired-end reads were aligned to hg19 reference genome with Bowtie2 (v2.3.4.1) (29) with options: --local --dovetail --minins 50 --maxins 600 --no-mixed --no-discordant. PCR duplicates were removed with Picard (v1.88). Peak-calling was performed with MACS2 (v2.1.1) with default parameters. For CUT&RUN, normal goat IgG sample was used as control, and for ChIP-seq, the input sample was used as a control during peak-calling. Peak annotation was performed with Homer (v4.10) (30). Peak comparison was performed with bedtools (v2.27.1) “intersectBed” function (31). When generating bigWig files, the library size scaling factor was applied.

Pathway enrichment analysis was performed using Enrichr (http://amp.pharm.mssm.edu/Enrichr/) (32, 33), and results from WikiPathways 2019 Human are presented.

#### RNA-seq and 4sU-seq analysis

Paired-end reads were aligned to hg19 reference genome with STAR (V2.5.0c) (34). For 4sU-seq, reads were also aligned to sacCer3 reference genome, and the number of reads aligned to *S. cerevisiae* was used as a scaling factor. Differential expression analysis was performed using DESeq2.

#### Metaplot and heat map

To plot metagene plots and heat maps, we selected Refseq genes that (1) have FPKM >5 in DLD1 WT RNA-seq and (2) have no overlapping transcripts within 5 kb upstream or downstream of the gene. 4544 genes were used for plotting. Metagene plot and heat map were plotted with deepTools (v3.1.0) (35). For ChIP-seq and CUT&RUN, a 10-bp bin size was used and for RNA-seq and 4sU-seq, a 100-bp bin size was used. The signal was normalized to the library size.

#### Calculating the pausing index

Total pol II ChIP-seq data were used to calculate the pausing index. We selected genes that (1) have an FPKM >5 in DLD1 WT RNA-seq, (2) are longer than 300 bp, and (3) have no overlapping transcripts within 1 kb upstream or downstream of the gene. 5939 genes were used for calculation. The TSS region is defined as 100 bp upstream to 300 bp downstream of TSS, and the gene body region is defined as 300 bp downstream of the TSS to TES. Pol II ChIP-seq reads within TSS and gene body were counted with bedtools (v2.27.1) “bedtools coverage” function, and FPKM was calculated. The pausing index was defined as log2[(TSS_FPKM +1)/(genebody_FPKM +1)].

## Data availability

All next-generation sequencing data have been deposited to Gene Expression Omnibus under the accession number GSE145368. Mass spectrometry raw files are accessible under MassIVE ID: MSV000085926 and ProteomeXchange: PXD020796.

## Conflict of interest

The authors declare that they have no conflict of interest with the contents of this article.

## Supporting information

JBC_JBC/2020/015876_1
