# Supplementary material for: Elongin A regulates transcription in vivo through enhanced RNA polymerase processivity
Source: J Biol Chem. 2020 Dec 13;296:100170. doi: 10.1074/jbc.RA120.015876 (PMC7948402; doi:10.1074/jbc.RA120.015876)
Supplement: Supporting Figures [file mmc2.pdf]

Supporting Figure Legend

Fig S1.

(**A**) Comparison of Elongin A peaks (left) and Elongin A occupied genes (right) identified by CUT&RUN and ChIP-seq. (**B**) Western blot for the Elongin A antibody used in this study. Lysate from DLD1 cells was used. Arrow indicates position of Elongin A band. (**C**) Pathway analysis of Elongin A-occupied genes. Top ranked categories are shown here. (**D**) Representative screenshots showing co-localization of Elongin A Cut&Run and ChIP-seq at some highly transcribed regions. (**E**) Representative screenshots showing Elongin A Cut&Run signal at transcribed regions and its co-localization with RNA pol II and RNA-seq signal. (**F**) Meta-plot of average Elongin A Cut&Run signal on 4,544 expressed, non-overlapping Refseq genes near TSS.

Fig S2.

(**A**) Representative screenshots showing Elongin A Cut&Run signal at intergenic regions and its co-localization with RNA pol II and nascent transcripts. Note the bi-directional transcript from intergenic Elongin A peak. (**B**) Detailed annotation of Elongin A Cut&Run peaks. (**C**) Analysis of the 2,545 intergenic Elongin A peaks. A large portion of these peaks co-localize with RNA Pol II or H3K27ac but not H3K27me3. (**D**) Top hits from Elongin A mass spectrometry. (**E**) Top: Gene ontology analysis of the top hits identified in mass spectrometry. Bottom: PAF1 pull down in DLD1 cells showing the interaction between PAF1 and Elongin A. Note that Flag-tagged Elongin A is slightly larger than endogenous Elongin A.

Fig S3.

(**A**) Elongin A expression level in control (siNS) and Elongin A-depleted (siELOA) cells quantified by RT-qPCR. (n=3; error bar shows standard deviation). (**B**) Representative screen shots showing change in RNA Pol II upon Elongin A depletion. (**C**) Meta-plot of average ChIP-seq signal (2nd replicate) on 4,544 expressed, non-overlapping RefSeq genes in control (siNS) and Elongin A-depleted (siEloA) cells. (**D**) Heatmaps of RNA pol II signal (2nd replicate) on 4,544 expressed, non-overlapping RefSeq genes. Genes were ranked by their expression levels (high to low). (**E**) Meta-plot of RNA Pol II ChIP-seq on 2,545 intergenic Elongin A peaks. Plots were centered on the summit of Elongin A peaks.

Fig S4.

(**A**) Heatmap of differentially expressed genes (FC>1.5, q-value < 0.1) in control and Elongin A-depleted cells by RNA-seq. (**B**) Plot showing gene fold change in control and Elongin A depleted cells by 4sU-seq. (**C**) Meta-plot of 4sU-seq signal in control (siNS) and Elongin A-depleted cells (siEloA) (2nd replicate). (**D**) Heatmaps of $\log_2$ fold-change at each time point compared with 0 min. Note that negative values (blue) indicate clearance of reads. 4,544 expressed, non-overlapped refseq genes were ranked by length (short to long) (2nd replicate). (**E**) Meta-plot of $\log_2$ fold- change at each time point compared with 0 min. (2nd replicate).

Fig S1

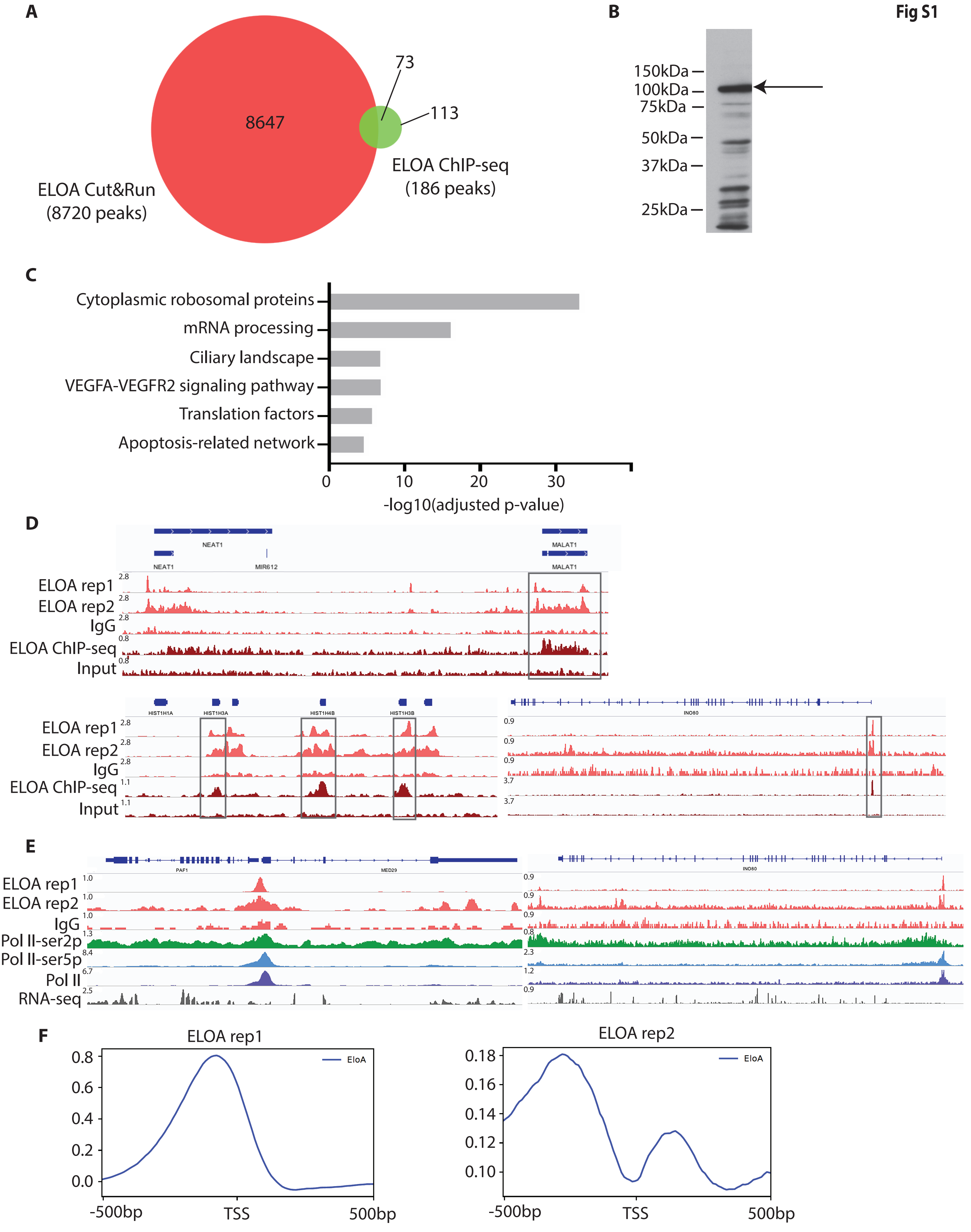
A
8647
73
113
ELOA Cut&Run
(8720 peaks)
ELOA ChIP-seq
(186 peaks)
B
150kDa
100kDa
75kDa
50kDa
37kDa
25kDa
C
Cytoplasmic robosomal proteins
mRNA processing
Ciliary landscape
VEGFA-VEGFR2 signaling pathway
Translation factors
Apoptosis-related network
0
10
20
30
-log10(adjusted p-value)
D
NEAT1
MIR612
MALAT1
HIST1H1A
HIST1H3A
HIST1H4B
HIST1H3B
INO80
ELOA rep1
ELOA rep2
IgG
ELOA ChIP-seq
Input
E
PAF1
MED29
INO80
ELOA rep1
ELOA rep2
IgG
Pol II-ser2p
Pol II-ser5p
Pol II
RNA-seq
F
ELOA rep1
ELOA rep2
EloA
0.8
0.6
0.4
0.2
0.0
0.18
0.16
0.14
0.12
0.10
-500bp
TSS
500bp

Fig S2

A

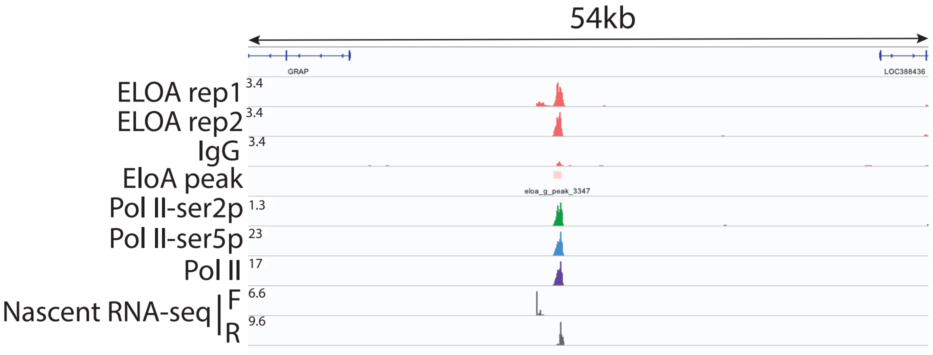

B

	Number of peaks	Percentage
Promoter	1324	15.20%
Gene body	4545	52.10%
TTS	306	3.50%
Intergenic	2545	29.20%
**TOTAL**	**8720**	

C

	Number of intergenic peaks colocalized	Percentage
Pol II	1417	55.70%
H3K27ac	720	28.30%
H3K27me3	24	0.01%

D

Accession number	Name	Peptide number	Molecular Weight (kDa)	Description
Q14241	ELOA	73	94.9	*Elongin complex*
Q15370	ELOB	10	13.1	
Q15369	ELOC	9	12.5	
Q8N7H5	PAF1	39	55.4	*PAF1 complex*
Q6PD62	CTR9	65	133.4	
Q6P1J9	CDC73	47	60.5	
Q8WVC0	LEO1	20	75.4	
Q9GZS3	WDR61	17	33.6	
P24928	RPB1	84	217.0	*Pol II subunits*
P30876	RPB2	61	133.8	
Q7KZ85	SPT6	82	198.9	*Pol II elongation*
O00267	SPT5	46	120.4	
Q9UHB7	AFF4	38	127.4	
Q9Y5B9	SPT16	38	119.8	*FACT complex*
Q08945	SSRP1	23	81.0	
Q8N1G1	REXO1	40	131.4	*mRNA processing*
O43290	SNUT1	33	90.2	
O94906	PRPF6	34	102.4	
O15042	U2SURP	25	118.2	
Q9UMS4	PRP19	14	55.1	
O43395	PRPF3	20	77.5	
Q8N201	INT1	28	244.1	*Integrator complex*
Q9NVH2	INT7	14	104.6	
Q96HW7	INT4	11	108.1	
Q96CB8	INT12	7	48.8	
Q9H0H0	INT2	10	134.2	

E

GO term	FDR
mRNA processing	4.67E-22
mRNA metabolic process	7.45E-21
RNA splicing	3.69E-20
RNA processing	2.78E-19
Transcription elongation	8.80E-19

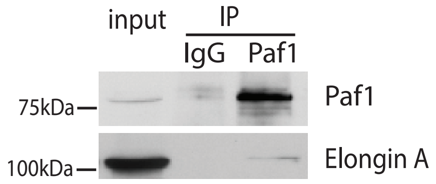

Fig S3

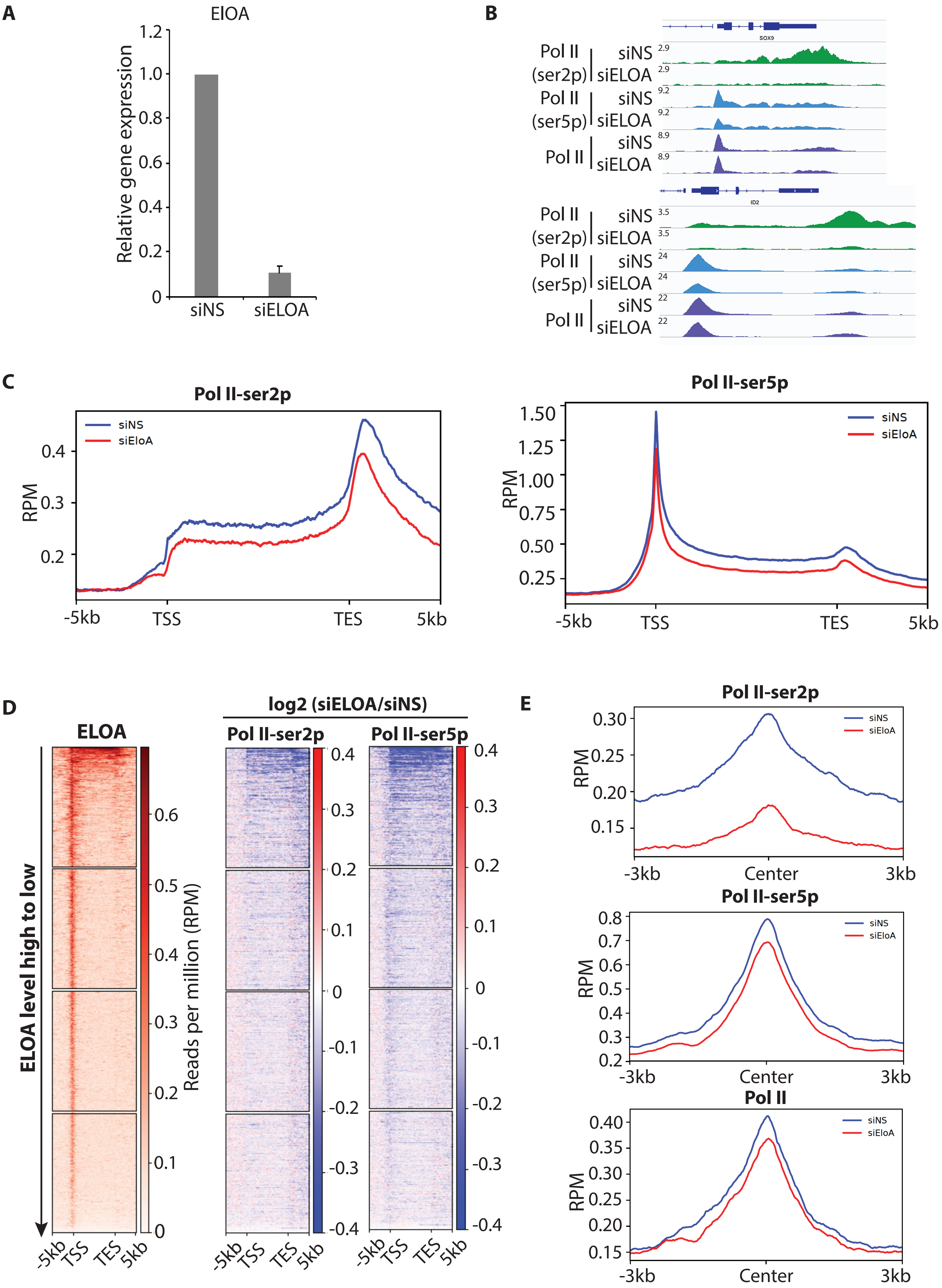

A
ElOA
Relative gene expression
1.2
1.0
0.8
0.6
0.4
0.2
0
siNS
siELOA
B
SOX9
Pol II (ser2p)
siNS
siELOA
Pol II (ser5p)
siNS
siELOA
Pol II
siNS
siELOA
2.9
9.2
8.9
ID2
3.5
24
22
C
Pol II-ser2p
siNS
siEloA
RPM
0.4
0.3
0.2
-5kb
TSS
TES
5kb
Pol II-ser5p
1.50
1.25
1.00
0.75
0.50
0.25
D
ELOA
ELOA level high to low
Reads per million (RPM)
0.6
0.5
0.4
0.3
0.2
0.1
0
log2 (siELOA/siNS)
Pol II-ser2p
Pol II-ser5p
0.4
0.3
0.2
0.1
0
-0.1
-0.2
-0.3
-0.4
E
Pol II-ser2p
0.30
0.25
0.20
0.15
-3kb
Center
3kb
Pol II-ser5p
0.8
0.7
0.6
0.5
0.4
0.3
0.2
Pol II
0.40
0.35
0.30
0.25
0.20
0.15

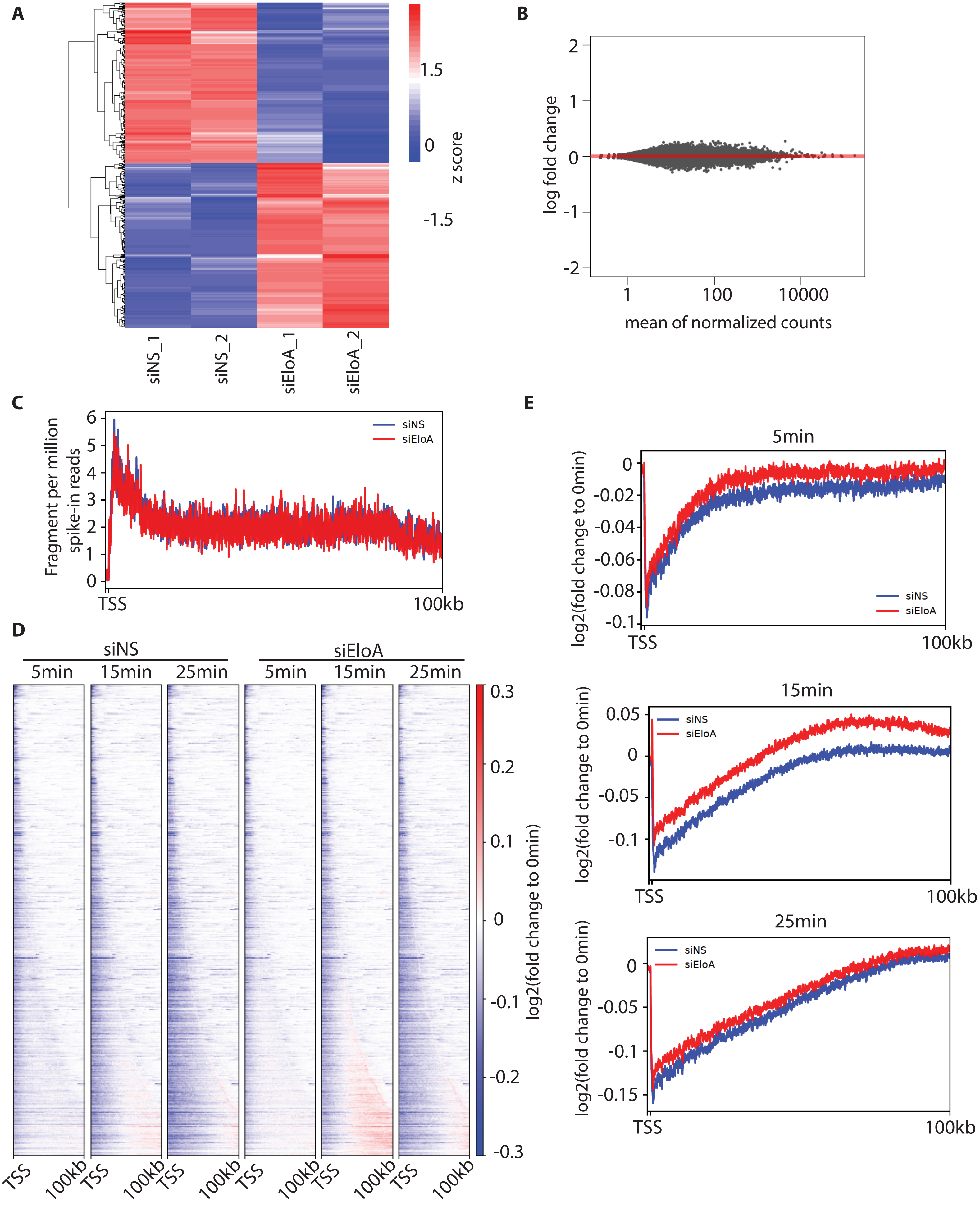
A
1.5
0
-1.5
z score
siNS_1
siNS_2
siEloA_1
siEloA_2
B
log fold change
mean of normalized counts
2
1
0
-1
-2
1
100
10000
C
Fragment per million spike-in reads
siNS
siEloA
TSS
100kb
D
siNS
siEloA
5min
15min
25min
0.3
0.2
0.1
0
-0.1
-0.2
-0.3
log2(fold change to 0min)
E
5min
15min
25min
log2(fold change to 0min)
0
-0.02
-0.04
-0.06
-0.08
-0.1
0.05
-0.05
-0.15
TSS
100kb
